# Mobile Screening Unit (MSU) for the Implementation of the ‘Screen and Treat’ Programme for Cervical Cancer Prevention In Pune, India

**DOI:** 10.31557/APJCP.2021.22.2.413

**Published:** 2021-02

**Authors:** Smita Joshi, Richard Muwonge, Vinay Kulkarni, Eric Lucas, Sanjeevani Kulkarni, Seema Kand, Mahesh Mandolkar, Mufid Baig, Sudhakar Wankhede, Kavita Surwase, Dilip Pardeshi, Partha Basu, Sankaranarayanan Rengaswamy

**Affiliations:** 1 *Prayas, Amrita Clinic, Athawale Corner, Karve Road, Deccan Gymkhana, Pune 411 004, India. *; 2 *Screening Group, Early Detection and Prevention Section, International Agency for Research on Cancer, WHO, Lyon, France. *; 3 *Research Triangle Institute, International-India, Commercial Tower, Pullman Hotel Aerocity, New Delhi, India. *

**Keywords:** Mobile screening unit, cervical cancer, screening, thermal ablation

## Abstract

**Objectives:**

We are reporting the evaluation of an opportunistic point of care cervical cancer screening initiative in Pune, India using a mobile screening unit (MSU).

**Methods:**

We conducted 290 cervical cancer screening outreach clinics in the MSU. Screening was performed by trained nurses/ health care providers using visual inspection with 5% acetic acid (VIA). Screen positive women when eligible were treated by thermal ablation during the same sitting. Women with large lesions not eligible for treatment with thermal ablation were referred for colposcopy and treatment.

**Results:**

A total of 10, 925 women were screened between Nov 2016 and June 2019 in 290 outreach clinics in the MSU. The overall screen positivity was 6.6% (95% CI 6.1, 7.0) with a declining trend over time. A total of 304/717 (42.4%, 95% CI 38.7, 46.1) women received treatment with thermal ablation. About 3.6% (11/304) reported minor side effects and 1.6% (5/304) reported lower abdominal pain and all of them subsided after treatment. Among the 413 women who were advised colposcopy, only 84 (20.33%) women underwent the procedure. Of these 84 women, 64 (76.19%) had normal colposcopy/ histopathology, 7 (8.33%) had CIN1, 2 (2.38%) had CIN 2, 9 (10.71%) had CIN 3 disease and 2 (2.38%) women were diagnosed with invasive cancer.

**Conclusion:**

MSUs are useful for providing cervical cancer screening services, using the ‘screen and treat’ strategy. Thermal ablation is safe in the field clinics. Additional efforts are needed to improve the compliance for referral of those with large lesions requiring additional visits.

## Introduction

Cervical cancer is the fourth most common cancer among women (Arbyn et al., 2020). In 2018, the age-standardised incidence of cervical cancer was 13.1 per 100 000 women globally (Arbyn et al., 2020). Cervical cancer is a major public health problem specially among women from the low- and middle-income countries. India accounts to about a fifth of the global burden of cervical cancer and has contributed 97 000 cases and 60 000 deaths in 2018 (Arbyn et al., 2020). Screening and vaccination are two powerful tools that are currently available for preventing cervical cancer and have the potential to practically eliminate cervical cancer. Vaccination of adolescent girls is very promising especially in the countries that do not have screening programs in place. However, cervical cancer screening remains the only option to prevent cervical cancer for unvaccinated adult women. High coverage, rapid scale-up of HPV vaccination to 80-100% girls globally by 2020 could avert 6.7-7.7 million cases of cervical cancer but more than half will be averted only after 2060 (Simms et al., 2019) and for the nearer term impact, we need high coverage cervical cancer screening (Simms et al., 2019).

Many European countries have been implementing organized screening programs for cervical cancer screening and have seen the decline in incidence and mortality due to cervical cancer (Arbyn et al., 2009). Organized, population-based screening programs are more effective in reducing cancer incidence and mortality (IARC, 2005). However, organization of such programs has not been possible in many developing countries. Government of India (GOI) developed guidelines for cervical cancer screening way back in 2006 (Government of India, 2006) but cervical cancer screening is not yet included in most of the public health services which are already overwhelmed by the patient oriented services and preventive services are sort of neglected. 

The World Health Organization (WHO) has called for a global action to eliminate cervical cancer within this century. This elimination call includes HPV vaccination of adolescent girls as well as screening of women aged 30 to 49 with a high-performance test and early detection and treatment of pre-cancers as well as invasive cancers (WHO, 2020). The currently available high-performance test is the HPV test but it is expensive. Visual inspection with 3-5% acetic acid (VIA) test is an alternative to HPV testing or traditional cytology screening. VIA has comparable sensitivity to that of cytology; nurses or health care providers can be trained to perform VIA, its results are available immediately after a minute and this provides an opportunity to treat screen-positive women with small, eligible lesions during the same sitting (Sankaranarayanan et al., 2005). VIA has been recommended as an alternative screening test when resources for the HPV test are not available (Santesso et al., 2016). WHO has also published simplified screening and treatment protocols (including screen-and-treat approach) that can be implemented in the low and middle-income countries with limited resources and infrastructure (Santesso et al., 2016).

WHO’s protocol using ‘screen with VIA and treat with ablation (if eligible)’ has opened up an opportunity to provide cervical cancer screening services practically at the door step in densely populated urban as well as rural areas for women in the low and middle-income strata. Despite the high risk of disease, cervical cancer screening coverage in India is very low, estimated to be about 5% (Gakidou et al., 2008) to 30% (van Dyne et al., 2019).

In an attempt to increase the opportunity for women to get screened and increase awareness about cervical cancer screening, we have a programme that provides opportunistic cervical cancer screening using the ‘screen and treat’ strategy in Pune district, Maharashtra, India using a mobile screening unit (MSU). Use of an MSU for screening for chronic diseases is not new and was recommended for resource limited settings about 50 years ago (Grant, 1965). MSUs can provide cancer screening services in the community settings and increase the access to early detection services. MSUs can overcome some of the barriers, save money and time, and this is particularly important for asymptomatic women who cannot afford to lose their daily wages to travel long-distances to the clinic settings. We are reporting the findings of our cervical cancer screening initiative using the MSU. 

## Materials and Methods

Prayas’s Ethics Committee has exempted program analysis of these data. Cervical cancer screening using VIA and immediate treatment using thermal ablation was provided in the MSU (Force Motors, Traveller & BSIII) customized to provide the services. The MSU was designed with the front compartment for the sitting arrangement for staff and storage underneath. An elongated compartment at the back was designed with an examination bed, light source for visual examination, a wall-mounted clock visible to the nurse while performing VIA, a platform to keep material/ instruments for screening and treatment, sitting and storage area and a small wash basin with a foot operated tap. The list of material carried in the vehicle is listed in Appendix 1. The vehicle was also fitted with a generator (900 VA) for providing electricity for the light source, fans, air-conditioner and treatment with thermal ablation. We initially used WisapTM coagulator (Germany) and then LigerTM (UT, USA) battery-operated coagulator for thermal ablation. 

Cervical cancer screening, ablative treatment and additional services (colposcopy, biopsy and excisional treatment whenever required) were provided free of cost. A team of 7 staff members (a medical officer for overall supervision, a community coordinator, a counsellor, a data entry operator, 2 nurses/nurse assistant/trained health care worker and a driver) travelled in the MSU. A WhatsApp group was created for the programme coordination, communication, for the project in-charge to give uniform instructions to the field staff and for the staff to provide daily update of the women screened (without divulging personal information), treated with ablation, referred to the clinic and for requesting any supplies needed in the field. Women with large lesions who could not be treated in the vehicle or those who were menstruating at the time of the screening visit in the MSU were referred/called for colposcopy, biopsy and treatment (ablation or excision) at our project clinic on Fridays. 

The screening clinics were arranged in and around Pune city within 60 to 70 Km distance. They were arranged in collaboration with the local non-governmental organizations (NGOs), self-help groups, women’s organizations/ groups in the community setting. Group meetings were conducted with women at the local community centre for awareness preferably on a day prior to the screening clinic and the day of screening clinic was informed to the women. When such a meeting was not possible, group meeting was conducted on the day of screening. Whenever a meeting hall was available, a short 10-minute film (https://www.youtube.com/watch?v=z2d-8L-NsBU) for awareness was projected (using a laptop, speakers and an LCD projector). After obtaining a written informed consent (or a thumb impression) of women who were non-pregnant, between 30 and 60 years of age and who had not undergone a hysterectomy, a structured questionnaire was completed to document the demographic information. Menstruating women were not excluded from screening. On an average 40-50 women were scheduled to be screened in a day.

The IARC manual was used to train the staff in screening with VIA (Sankaranarayanan and Wesley, 2003). Screening and treatment with thermal ablation were provided by trained nurses/ health care provider. After exposing the cervix with the help of a bi-valve, self-retaining Cusco or Graves speculum in a modified lithotomy position, 3-5% dilute acetic acid was applied with a sterile cotton swab and result was noted after one minute. VIA negative women were advised a repeat test after 3 years. If VIA was positive, immediate ablative treatment with thermal ablation was advised when the eligibility for ablation was met, that is, when the lesion involved less than 3 quadrants of the cervix, there was no extension of the lesion into endocervical canal or vagina, when the lesion was on the ectocervix with the squamo-columnar junction (SCJ) seen in its entirety, and when there was no suspicion of an invasive cancer. Since this was a ‘screen and treat programme’, treatment to screen positive women with eligible lesions was provided with thermal ablation (WisapTM coagulator,105 degree centigrade for 45 seconds or LigerTM coagulator, 100 degree centigrade for 40 seconds) without a biopsy. Multiple overlapping applications were given for large lesions in order to ablate the entire transformation zone. A printed card with instructions in local language was given to women after treatment with thermal ablation and they were asked to contact the clinic for any untoward symptoms. Women who were treated by thermal ablation were called for a clinic visit after 3 months during which a simple speculum examination was done to assess the healing of the cervix and the questionnaire about side effects/ complications if any after treatment was completed. 

A data entry operator entered data on-site using a laptop and a portable internet device into a digital platform designed by the screening group of IARC. The computer-generated report was printed and given to the woman along with a brochure for cervical cancer prevention awareness. 

Women with large lesions, not eligible for ablation were referred to the clinic where they underwent colposcopy, directed biopsy if needed and ablative treatment or loop electro-excision procedure (LEEP) aka large loop excision of transformation zone (LLETZ). LEEP was performed under local anaesthesia at our clinic. Women diagnosed with invasive cancer or women with extensive lesions probably requiring a cold knife conization or LEEP under general anaesthesia were referred appropriately. 

Biomedical waste was collected in 3 different plastic containers/ dustbins (one each for used specula, cotton sticks and gloves) lined with disposable plastic bags. These plastic bags were sent to the programme clinic for further processing after tying the plastic bags appropriately. At the clinic, plastic bags containing cotton sticks and gloves were disposed of as per the local biomedical waste disposal guidelines and used specula were disinfected in 0.5% sodium hypochlorite solution for 20 minutes prior to washing with soap and water and autoclaving. 

**Table 1:** Women Characteristics by Screening Period

Characteristics	Baseline screening period			Total
	November 2016 to October 2017 (Year I)	November 2017 to October 2018 (Year II)	November 2018 to June 2019 (Year III)	
	n (%)	n (%)	n (%)	n (%)
Women screened	3,537	4,566	2,822	10,925
Age				
21-29	6 (0.2)	8 (0.2)	2 (0.1)	16 (0.1)
30-49	2,945 (83.3)	3913 (85.7)	2403 (85.2)	9261 (84.8)
50-64	586 (16.6)	645 (14.1)	417 (14.8)	1,648 (15.1)
Marital status				
Unmarried	6 (0.2)	8 (0.2)	5 (0.2)	19 (0.2)
Married	2,805 (79.3)	4,113 (90.1)	2,570 (91.1)	9,488 (86.8)
Widowed	655 (18.5)	395 (8.7)	208 (7.4)	1,258 (11.5)
Separated	70 (2.0)	50 (1.1)	39 (1.4)	159 (1.5)
Total number of pregnancies				
None	71 (2.0)	77 (1.7)	61 (2.2)	209 (1.9)
1	230 (6.5)	344 (7.5)	245 (8.7)	819 (7.5)
2-3	2,165 (61.2)	2,969 (65.0)	1,837 (65.1)	6,971 (63.8)
4+	1,071 (30.3)	1,176 (25.8)	679 (24.1)	2,926 (26.8)
Date of last menstruation				
<12	3,449 (97.5)	4,494 (98.4)	2,776 (98.4)	10,719 (98.1)
>12	88 (2.5)	72 (1.6)	46 (1.6)	206 (1.9)

**Table 2:** Year Wise Screening with VIA and Treatment with Thermal Ablation

	Nov 2016 to Oct 2017 Year I	Nov 2017 to Oct 2018 Year II	Nov 2018 to June 2019 Year III	Total
Screening camps - n	89	125	76	290
Women screened with VIA	3,537	4,566	2,822	10,925
Visibility of squamocolumnar junction				
Fully - n	2,106	2,916	1,718	6,740
%	59.5	63.9	60.9	61.7
Partially - n	517	422	312	1,251
%	14.6	9.2	11.1	11.5
Not visible - n	914	1,228	792	2,934
%	25.8	26.9	28.1	26.9
Women positive on VIA - n	250	316	151	717
%	7.1	6.9	5.4	6.6
95%CI	6.2 -8	6.2 -7.7	4.5 -6.2	6.1 -7
Women positive on VIA who received				
thermal ablation treatment - n	102	129	73	304
%	40.8	40.8	48.3	42.4
95%CI	34.6 -47.2	35.4 -46.5	40.1 -56.6	38.7 -46.1
Women positive on VIA who received thermal ablation treatment on the same day of screening				
n	93	123	70	286
%	91.2	95.3	95.9	94.1
95%CI	83.9 -95.9	90.2 -98.3	88.5 -99.1	90.8 -96.5
Invasive cancers	0	1	1	2

VIA, visual inspection with acetic acid

## Results

The initiative was implemented from November 2016 to June 2019 and a total of 10,925 women were screened with 3,537 women in Year I, 4,566 women in Year II and 2,822 women in Year III. Table 1 provides the screening period, the number of women screened per year and their demographic data. Overall, 9261/10,925 (84.8%) women were between 30 to 49 years of age and 9488/10,925 (86.8%) women were married. Most women (6,971/10,925, 63.8%) reported 2-3 pregnancies (number of times they became pregnant including abortions) and 2,926/10,925 (26.8%) women reported 4+ pregnancies. 

Table 2 provides year-wise screening clinics, number of women screened per year and screening findings. We conducted 89, 125 and 76 screening clinics in Year I, II and III respectively with a total of 290 clinics and screened 10,925 women with the average of 38 women screened per clinic. Once planned, none of the screening clinics were cancelled on the day of the clinic, although sometimes they had to be postponed and participation rates were sometimes affected by heavy rainfall or cultural issues such as festivals. Except for one outreach clinic when the gloves had to be purchased from a nearby medical facility, our team did not face any problems with the supplies. Of the 10,925 women screened, the SCJ was fully visible in 6740 (61.7%) women, partially visible in 1251 (11.5%) women and not visible in 2934 (26.9%) women. VIA positivity was 7.1% (95% CI 6.2, 8.0), 6.9% (95% CI 6.2, 7.7) and 5.4% (95% CI 4.5, 6.2) during the consecutive 3-year period. Overall VIA positivity was 717/10,925 (6.6%, 95% CI 6.1, 7.0). Of the total 717 women who were VIA positive, 304 (42.4%, 95% CI 38.7, 46.1) received ablative treatment and the majority (286/304, 94.1%, 95% CI 90.8, 96.5) received it on the same day of screening. None of them refused treatment during the screening visit in the MSU. Among the remaining 413 women who were advised colposcopy, only 84 women (20.33%) visited the colposcopy clinic. Of these 84 women, 64 (76.19%) had normal colposcopy/ histopathology, 7 (8.33%) had CIN1, 2 (2.38%) had CIN 2, 9 (10.71%) had CIN 3 disease and 2 (2.38%) invasive cancers were detected among them. Thus, among those who underwent further investigations at the clinic, 13/84 (15.47%) had CIN 2+ disease. Women with invasive cancer were referred appropriately and women with CIN diagnosis were treated by ablation or LEEP. 

Following treatment with thermal ablation, mild pain/ cramps were reported by 6/304 (2.0%) women, mild bleeding and foul-smelling discharge by 2/304 (0.7%) women each and one woman complained of menorrhagia (1/304, 0.3%). Lower abdominal pain was reported by 5/304 women (1.6%) which subsided after syndromic management. [Data not shown]. 

## Discussion

We have demonstrated the feasibility of using an MSU over a period of two and a half years for cervical cancer screening in Pune, India. Use of MSU has been reported earlier specially for breast and cervical cancer screening across low, middle as well as high-income countries (Greenwald et al., 2017). However there is only report on breast, cervical and oral cancer screening from India (Kumar et al., 2011). Almost all the publications on MSU use for cervical cancer screening have mentioned either Pap testing or an HPV test and these studies were done prior to the WHO’s recommendation of ‘screen and treat’ strategy. With the goal of cervical cancer elimination within this century and with the increasing recognition of the fact that we need innovative approaches to improve screening coverage and that ‘one size does not fit all (Olsson and Lau, 2015; Printz, 2017), use of the MSU provides an opportunity to reach the unscreened population in remote areas. Involvement of the private medical practitioners to provide affordable cervical cancer screening (Shikha et al., 2020) and including cervical cancer screening in the family planning clinics are some of the other options (Mignot et al., 2019) for improving screening coverage in India. It is also important that such programs are sustained and scaled-up to reach maximum possible number of women in the country. If equipped with facilities to treat any emergencies such as anaphylaxis, MSU can also be used for vaccinating the girls against HPV as well as screening the mothers for breast and cervical cancer. 

The instant mobile messaging of WhatsApp available for smartphones was very useful and effective for coordination, logistics management and communication with the field staff. WhatsApp has recently started getting attention in healthcare systems coordination (Pahwa et al., 2018; Dorwal et al., 2016).

Ablative treatment in a ‘screen and treat’ program can be offered to women who fulfil certain eligibility criteria and visibility of the SCJ in its entirety is an essential element (WHO, 2013). In our programme, SCJ was fully visible or partially visible in more than 70% of the women. This is important because the sensitivity of VIA decreases in older population and also because the treatment decision is made based on the SCJ visibility. The overall VIA positivity in our project was 6.6% with a declining trend from 7.1% to 5.4%. The decline in positivity possibly indicates increased confidence of nurses in identifying the abnormal lesions. VIA positivity in different projects in India has ranged from 5.4% (Shikha et al., 2020) to 10.75% (Poli et al., 2015) and there was a lot of variation from site to site. In a large demonstration project in 6 African countries, VIA positivity ranged from 5.7% to 28.0% (WHO, 2012) and it varied across the countries. We did not see such a wide variation during the project period as we ensured adequate training, re-orientation and supervision of the nurses performing VIA. 

Another important feature of our initiative is the use of thermal ablation for the treatment of screen positive women. A randomised controlled trial in Zambia that compared cryotherapy with thermal ablation has reported similar success rates of thermal ablation when compared to that of cryotherapy (Pinder et al., 2020). In our study, thermal ablation treatment was safe, well tolerated and without significant side effects when used by the nurses alone in the MSU. We have previously reported use of the thermal ablation (cold coagulator) for the treatment of cervical intraepithelial neoplasia in HIV-infected women (Joshi et al., 2013) and also among female sex workers (Joshi et al., 2015). A meta-analysis of studies that used thermal ablation suggests the overall response rate for biopsy proven CIN2+ disease to be 93.8% (Randall et al., 2019). We have systematically collected data on safety of thermal ablation in our program. Cold coagulation is actually a misnomer and the WHO guidelines have used the terminology as ‘thermal ablation’ (WHO, 2019). About 42% of the screen positive women received same day treatment in the MSU and this enforces the feasibility and acceptability of the screen-and-treat approach. This also underscores the benefits associated with the ‘screen and treat’ program. 

In our initiative, among the screen positive women, 54% women received treatment/ completed diagnostic investigations whereas 46% defaulted therefore we do not know their disease profile and some of them might be having high-grade disease as they could not be treated with thermal ablation in the field clinics. Very few (20%) women who were screen positive and could not be treated in the MSU visited our clinic, 15% of them had > CIN 2 disease. We did not try to investigate the factors associated with loss-to-follow-up but they could be limited time, unaffordable transportation costs (The Lancet Oncology, 2020) and poor knowledge (Siddharthar et al., 2014). And what is uniquely lacking in India is public information campaign about the need for screening. Additional research is also needed in India to improve the adherence to colposcopy visits and appropriate treatment in the screen and treat programmes. The population-level cervical cancer programme in Zambia has shown high level of compliance to further diagnosis/ treatment and in their large programme that screened over 100,000 women, 70.3% of the screen positive women received ablative or excisional treatment (Parham et al., 2015).

At this stage, the novel coronavirus pandemic has engulfed the entire world and it appears that we will have to wait, watch and adapt until the immediate and long-term effects of this pandemic fully materialise (The Lancet Oncology, 2020). We hope that cervical cancer prevention efforts are not stalled after the pandemic recedes, otherwise there will be substantial increase in the cervical cancer cases in the next 20 years. It is possible that countries that were planning to introduce an HPV test will have to re-think about its use and consider using VIA for cervical cancer screening which has considerable cost savings as compared to an HPV test and our experience of using the MSU will help them shape future interventions for cervical cancer prevention. 

*Disclaimer*

Where authors are identified as personnel of the International Agency for Research on Cancer/World Health Organization, the authors alone are responsible for the views expressed in this article and they do not necessarily represent the decisions, policy or views of the International Agency for Research on Cancer /World Health Organization.
